# The combination of cardiorespiratory fitness and muscle strength, and mortality risk

**DOI:** 10.1007/s10654-018-0384-x

**Published:** 2018-03-28

**Authors:** Youngwon Kim, Tom White, Katrien Wijndaele, Kate Westgate, Stephen J. Sharp, Jørn W. Helge, Nick J. Wareham, Soren Brage

**Affiliations:** 1 MRC Epidemiology Unit, University of Cambridge School of Clinical Medicine, Box 285 Institute of Metabolic Science, Cambridge Biomedical Campus, Cambridge, Cambridgeshire CB2 0QQ UK; 2 Department of Health, Kinesiology, and Recreation, College of Health, University of Utah, 250 South 1850 East Room 204, Salt Lake City, UT 84112 USA; 3 Department of Biomedical Sciences, Center of Healthy Aging, University of Copenhagen, Blegdamsvej 3, 2200 N, Copenhagen, Denmark

**Keywords:** Cardiorespiratory fitness, Grip strength, Mortality, UK Biobank

## Abstract

Little is known about the combined associations of cardiorespiratory fitness (CRF) and hand grip strength (GS) with mortality in general adult populations. The purpose of this study was to compare the relative risk of mortality for CRF, GS, and their combination. In UK Biobank, a prospective cohort of > 0.5 million adults aged 40–69 years, CRF was measured through submaximal bike tests; GS was measured using a hand-dynamometer. This analysis is based on data from 70,913 men and women (832 all-cause, 177 cardiovascular and 503 cancer deaths over 5.7-year follow-up) who provided valid CRF and GS data, and with no history of heart attack/stroke/cancer at baseline. Compared with the lowest CRF category, the hazard ratio (HR) for all-cause mortality was 0.76 [95% confidence interval (CI) 0.64–0.89] and 0.65 (95% CI 0.55–0.78) for the middle and highest CRF categories, respectively, after adjustment for confounders and GS. The highest GS category had an HR of 0.79 (95% CI 0.66–0.95) for all-cause mortality compared with the lowest, after adjustment for confounders and CRF. Similar results were found for cardiovascular and cancer mortality. The HRs for the combination of highest CRF and GS were 0.53 (95% CI 0.39–0.72) for all-cause mortality and 0.31 (95% CI 0.14–0.67) for cardiovascular mortality, compared with the reference category of lowest CRF and GS: no significant association for cancer mortality (HR 0.70; 95% CI 0.48–1.02). CRF and GS are both independent predictors of mortality. Improving both CRF and muscle strength, as opposed to either of the two alone, may be the most effective behavioral strategy to reduce all-cause and cardiovascular mortality risk.

**Electronic supplementary material:**

The online version of this article (10.1007/s10654-018-0384-x) contains supplementary material, which is available to authorized users.

## Introduction

Low cardio-respiratory fitness (CRF) is a strong predictor of numerous health outcomes, including mortality, not only in general adult populations [1] but also in obese [2], hypertensive [3, 4], or diabetic [5] adults. Poor muscular strength, another component of fitness, has also been indicated as an important marker of mortality [6, 7] as well as adverse health outcomes such as frailty and sarcopenia [8]. Recent clinical trials [9–12] have demonstrated that compared with improving either CRF or muscle strength alone, improving both CRF and muscle strength simultaneously led to more favorable changes on intermediate cardio-metabolic risk factors and functional status; these intermediate outcomes are important predictors for clinical end-points [13, 14]. However, the prior clinical trials [9–12] did not include clinical end-points, and it would, in fact, be nearly impossible to establish clinical trials with sufficient statistical power to do so. In contrast, large-scale prospective observational studies can provide such evidence. However, a few observational studies evaluating combined impacts of CRF and muscle strength in relation to mortality risk merely included data from highly select populations of men (i.e. men who were either hypertensive [15] or adolescent [16] at baseline). This limitation precludes the ability to draw robust conclusions for general adult populations of men and women. Given that current public health guidelines [17] recommend that men and women engage in both aerobic and muscle-strengthening activities across the whole lifespan, it is critical from clinical and public health standpoints to examine the combined impacts of CRF and muscle strength for mortality risk for a broader adult population.

The UK Biobank study is an ongoing prospective national cohort of over half a million middle-aged UK men and women. Data collection at baseline and repeat-assessment visit included submaximal stationary bike tests to assess CRF as well as grip strength (GS) to evaluate overall muscle strength [18–20]. This provides an opportunity to disentangle the interplay between CRF, muscle strength and mortality in general adult populations. Therefore, the purpose of this study was to explore the relative risk of mortality from all causes, cardiovascular disease (CVD) and cancer for CRF, GS and the combination of both.

## Methods

### Study design and participants

**Fig. 1:**
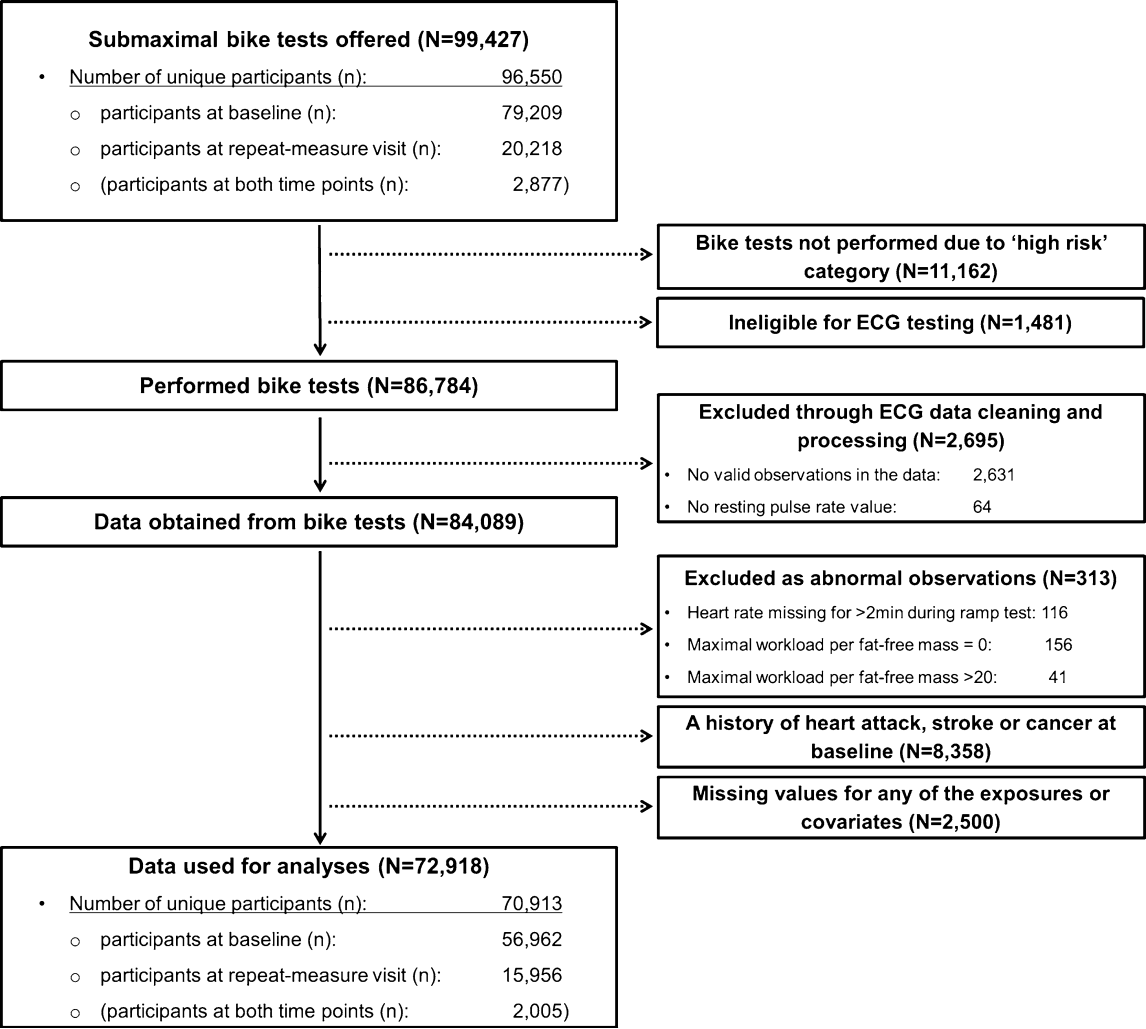
A flow diagram showing the number of data cases included or excluded at each stage. Note: “N” indicates numbers of total participants (i.e. participants who provided repeated measures are treated as separate data cases) and “n” indicates numbers of unique participants

Approximately 9.2 million adults who were within < 25 miles of one of 22 assessment centres across the UK and registered with the National Health Service were initially contacted for participation in the UK Biobank study. Between 2006 and 2010, > 500,000 participants underwent baseline data collection which included a wide variety of physical measurements and biological samples, as well as questionnaires on prevalent morbidities, socio-demographic factors, family history/early-life exposures, lifestyle, and environmental factors. Repeated assessments of the variables were carried out between 2012 and 2013 in a sub-sample of over 20,000 individuals. From 2009, the baseline protocol was extended to include submaximal stationary bike tests to assess CRF; this was offered to 96,550 participants (79,209 from baseline; 20,218 from the repeat-measures visit), totaling 99,427 measurements (2877 at both time points). More details about the UK Biobank methodology are provided elsewhere [21]; Fig. 1 provides an overview of participants included in the present analysis. All participants signed informed written consent prior to participation, and the UK Biobank protocol was approved by the North West Multi-Centre Research Ethics Committee.

### Exposures

#### Cardiorespiratory fitness

Prior to performing a submaximal exercise test on a stationary bike (eBike Comfort Ergometer, General Electric, firmware version 1.7), participants were categorized into one of five risk categories (S1 Material in Appendix) [22]. The risk categorization determined allocation to an individualized exercise protocol (S2 Material in Appendix), a methodology aimed at increasing the number of participants with exposure information whilst at the same time reducing the likelihood that participants experience any adverse medical events during the exercise test. Individuals with ‘minimal risk’ (n = 72,715) and ‘small risk’ (n = 11,257) carried out standard bike protocols, which consisted of (1) an initial 15-s seated-rest period, (2) a 2-min phase at constant power (30 watts for women; 40 watts for men), (3) a 4-min ramp phase with linear increases in power from their initial constant power to their individually assigned peak power (to 50 and 35% of predicted maximal workload for ‘minimal’ and ‘small’ risk, respectively), and (4) a 1-min recovery period. Individuals with ‘medium risk’ (n = 2812) cycled at the constant power level for 6 min. Participants were asked to cycle at 60 revolutions-per-minute (RPM) during all cycling phases. Individuals in the ‘high’ risk (n = 11,162) category only did a 2-min seated-rest assessment and were excluded from this analysis, as were those ‘ineligible’ for electrocardiograph testing (n = 1481).

Participants’ electrocardiograms were recorded at 500 Hz with a 4-lead electrocardiograph device (CAM-USB 6.5, Cardiosoft v6.51; two electrocardiograph electrodes on each upper limb) throughout the full test. The electrocardiograph signal was processed using the PhysioNet Toolkit [23] implementation of the SQRS algorithm [24], which applies a digital filter to the signal and identifies the distinctive downward slopes of QRS complexes. The resultant inter-beat-intervals were converted to beats-per-minute values, using “ihr” of the PhysioNet Toolkit [25] (restricting beat-to-beat heart rate changes to ≤ 10 bpm), after which linear interpolation was applied to derive heart rate at 1-s resolution. In addition, we implemented data cleaning and quality control procedures (S3 Material in Appendix). Linear regression was performed to predict workload from heart rate (S4 Material in Appendix); the established linear relationship was then extrapolated to age-predicted maximum heart rate [26] to estimate an individual’s maximal power (watts) as an indicator of CRF. Consolidation procedures were applied to obtain the most robust CRF estimate (S5 Material in Appendix). To account for differences in body size, CRF was expressed as maximal power per fat-free mass (kg) (i.e. body mass–fat mass), the latter measured using bio-impedance analysis (Tanita BC-418MA). Individuals with heart rate missing for > 2 min (50%) during the ramp phase (n = 116) or with maximum power of 0 (n = 156; a sign of the cycle ergometer/ECG acquisition system malfunctioning) or outliers with > 20 watts/fat-free mass (n = 41) were excluded from analyses.

#### Grip strength

GS was assessed once in each hand using a hydraulic hand dynamometer (Jamar J00105), which can measure isometric grip force and was calibrated by staff at the start of each measurement day. Each participant grasped the handle of the device in their right hand while sitting upright on a chair with their forearm on the armrest, and whilst maintaining a 90° angle of their elbow, squeezed the handle as strongly as possible for about 3 s. The same protocol was undertaken with the left hand. GS measures have good reliability and reproducibility [27]. For the current primary analysis, values from the two hands were averaged if available; otherwise, the value from a single hand was used in a small subsample (n = 204). Values of GS (kg) were also divided by fat-free mass (kg) to account for differences in body size.

### Outcomes

Mortality status was ascertained by linking the Biobank data with death records from the National Health Service Information Centre and the Scottish Morbidity Record. For the present analyses, we used mortality cases accrued until February 15th 2016. Mortality from CVD and cancer were classified according to the International Classification of Diseases-10 codes F01 and I00-I99, and C00-D48, respectively. The median follow-up period was 5.7 years (interquartile range 5.6–5.9 years).

### Covariates

The following variables were included as covariates in the analyses: sex, waist circumference (centimeters), ethnicity (White, mixed, Asian/Asian British, Black/Black British, other), smoking status (never, previous, current), employment (unemployed, employed), Townsend Deprivation Index (a composite score of employment, car ownership, home ownership and household overcrowding; based on postcode, with higher values indicating a higher degree of deprivation), alcohol consumption (never, previously, currently < 3 times/week, currently ≥ 3 times/week), processed/red meat consumption (days/week), beta-blocker use (yes, no), hypertension, and diabetes. Hypertension was defined as systolic/diastolic blood pressure ≥ 140/90 mmHg, a physician diagnosis of hypertension, and/or reported medication used to regulate blood pressure. Participants were considered having diabetes if they reported a physician diagnosis of diabetes or were taking glucose-lowering medication. Participants with a self-reported history of heart attack, stroke or cancer were excluded, resulting in a final sample of 70,913 participants (2005 with repeated measures) with no missing values included in the analyses (Fig. 1).

### Statistical analyses

Sex- and age-specific categories of CRF and GS were calculated based on tertiles of their baseline distributions to categorize individuals into either low, medium or high CRF/GS at both baseline and the repeated exposure assessment (S1 Table in Appendix). Cox regression, with age as the underlying time scale, was used to estimate associations of CRF and GS with mortality, including the categories of CRF and GS at both baseline and follow-up as time-updated covariates. Models were fitted with no adjustment (Model 1), adjustment for potential confounders (Model 2), and further adjustment for GS in models for CRF or for CRF in models for GS (Model 3). Parallel sets of models were performed using standardized variables (i.e. per 1-standard deviation increment) of CRF and GS. Interactions of CRF or GS with sex were tested. Joint associations of CRF and GS with mortality were estimated using low GS/low CRF as the common reference group; the multiplicative interaction between CRF and GS was tested. Log–log plots provided support for the proportional hazards assumptions. The following sensitivity analyses were performed: (1) a random-effects meta-analysis across the different individualized protocols to examine the impacts of protocol assignment on fitness-mortality associations, (2) an analysis after excluding mortality cases occurring during the first 2 years of follow-up to address reverse causality, and (3) an analysis with CRF and GS both normalized for body weight to examine whether different handling of the scaling for body size influences mortality associations. Analyses were performed in Stata/SE Version 14 (StataCorp LP, College Station, TX).

## Results

Table 1 summarizes characteristics of the participants across CRF and GS categories. Individuals with greater CRF or GS were more likely to be smokers, or alcohol drinkers, be employed, live in less deprived areas, and have no hypertension or diabetes at baseline. The Pearson correlation between CRF and GS was moderate (0.55) [28].

**Table 1:** Participants’ characteristics

	All	Cardiorespiratory fitness			Grip strength		
	(N = 72,918; n = 70,913)	Low (N = 24,267; n = 23,723)	Middle (N = 24,058; n = 23,394)	High (N = 24,593; n = 23,796)	Low (N = 27,500; n = 26,676)	Middle (N = 23,487; n = 22,871)	High (N = 21,931; n = 21,366)
Cardiorespiratory fitness, watts	188.4 (83.5)	128.2 (51.3)	184.4 (59.1)	251.6 (84.0)	188.6 (85.5)	190.0 (83.3)	186.3 (80.9)
Grip strength, kg	29.4 (10.7)	28.7 (10.7)	29.5 (10.8)	30.0 (10.5)	23.0 (8.5)	30.3 (8.9)	36.3 (10.2)
Fat-free mass, kg	53.2 (11.4)	52.7 (11.6)	53.1 (11.4)	53.7 (11.1)	54.2 (12.0)	53.2 (11.3)	51.9 (10.6)
Age, years	57.2 (8.2)	57.7 (8.4)	57.1 (8.2)	56.6 (8.0)	57.8 (8.3)	57.0 (8.1)	56.4 (8.1)
Sex, %							
Women	53.1%	53.4%	53.4%	52.4%	53.4%	52.8%	52.8%
Men	47.0%	46.6%	46.6%	47.6%	46.6%	47.2%	47.2%
Waist circumference, cm	89.6 (13.0)	90.6 (13.8)	89.5 (12.8)	88.6 (12.3)	92.7 (13.9)	89.2 (12.4)	86.1 (11.5)
Ethnicity, %							
White	92.5%	88.9%	93.0%	95.5%	91.9%	92.9%	92.8%
Others	7.5%	11.1%	7.0%	4.5%	8.1%	7.2%	7.2%
Smoking status, %							
Never	57.8%	60.1%	57.6%	55.8%	58.6%	57.6%	57.1%
Previously	34.1%	32.2%	34.6%	35.4%	34.0%	34.2%	34.0%
Currently	8.1%	7.8%	7.8%	8.8%	7.5%	8.2%	8.9%
Employment, %							
Unemployed	42.9%	46.9%	42.3%	39.3%	46.1%	41.7%	40.0%
Townsend deprivation index	− 1.35 (2.90)	− 1.14 (3.01)	− 1.39 (2.88)	− 1.52 (2.79)	− 1.16 (2.99)	− 1.40 (2.89)	− 1.53 (2.80)
Alcohol consumption, %							
Never	4.2%	5.8%	4.0%	2.7%	5.0%	4.0%	3.3%
Previously	3.0%	3.4%	3.1%	2.6%	3.5%	3.1%	2.5%
Currently (< 3 times/week)	48.0%	51.3%	48.4%	44.4%	50.5%	47.3%	45.7%
Currently (≥ 3 times/week)	44.8%	39.4%	44.5%	50.3%	41.1%	45.6%	48.5%
Processed/red meat consumption, days/week	0.89 (0.56)	0.92 (0.58)	0.88 (0.55)	0.86 (0.55)	0.91 (0.58)	0.88 (0.55)	0.87 (0.55)
Beta-blocker use, %	4.7%	3.6%	3.5%	6.8%	5.6%	4.5%	3.7%
Hypertension, %	51.5%	63.2%	48.4%	42.9%	53.6%	50.7%	49.7%
Diabetes, %	4.7%	6.5%	4.2%	3.5%	6.8%	4.2%	2.6%

Values are means (standard deviations) unless otherwise indicated. Age- and sex-specific cut-points were used to create categories of cardiorespiratory fitness and grip strength. Note: “N” indicates numbers of total participants (i.e. participants who provided repeated measures are treated as separate data cases) and “n” indicates numbers of unique participants

Table 2 shows associations of CRF and GS with all-cause, CVD and cancer mortality. Over 379,682 person-years of follow-up, there were 832, 177 and 503 deaths from all causes, CVD and cancer, respectively. Crude mortality rates from all causes, CVD and cancer were consistently lower in those with higher levels of CRF or GS. Interactions of each exposure with sex were not significant (p-values < 0.05), so associations were estimated for men and women combined. Compared with the lowest category of CRF, the hazard ratios (HR) of all-cause mortality were lower for the higher CRF categories (*p* for trend: < 0.0001), after adjustment for potential confounders. Additional adjustment for GS made almost no difference to the results. Every 1-standard deviation increase in CRF was associated with 23% (95% CI 13–31%) lower hazard of all-cause mortality. A meta-analysis across the different individualized protocols (sensitivity analysis) revealed similar inverse (although less linear) associations (S2 Table in Appendix). Analyses using CRF estimates only from the constant phase also found similar inverse associations (data not shown).

**Table 2:** Independent associations of cardiorespiratory fitness and grip strength with mortality from all causes, cardiovascular disease (CVD) and cancer

Mortality type	Comparisons	Number of deaths	Person-years of follow-up	Crude mortality rate per 100,000-person years	Hazard ratios (95% confidence interval)		
					Model 1	Model 2	Model 3
All-cause mortality		832	379,682	219.1			
	Categories of cardiorespiratory fitness						
	Low (reference)	368	125,940	292.2	1.00 (reference)	1.00 (reference)	1.00 (reference)
	Middle	253	126,043	200.7	0.74 (0.63, 0.87)	0.75 (0.64, 0.89)	0.76 (0.64, 0.89)
	High	211	127,700	165.2	0.65 (0.55, 0.78)	0.65 (0.56, 0.77)	0.65 (0.55, 0.78)
	*P for linear trend*				< 0.0001	< 0.0001	< 0.0001
	Per 1-SD increase in cardiorespiratory fitness				0.68 (0.60, 0.78)	0.77 (0.68, 0.87)	0.77 (0.69, 0.87)
	Categories of grip strength						
	Low (reference)	349	136,899	254.9	1.00 (reference)	1.00 (reference)	1.00 (reference)
	Middle	265	124,095	213.5	0.86 (0.73, 1.00)	0.88 (0.75, 1.04)	0.88 (0.75, 1.04)
	High	218	118,689	183.7	0.76 (0.64, 0.90)	0.80 (0.67, 0.96)	0.79 (0.66, 0.95)
	* P for linear trend*				0.001	0.014	0.010
	Per 1-SD increase in grip strength				0.91 (0.85, 0.98)	0.94 (0.87, 1.01)	0.93 (0.86, 1.01)
CVD mortality		177	379,682	46.6			
	Categories of cardiorespiratory fitness						
	Low (reference)	87	125,940	69.1	1.00 (reference)	1.00 (reference)	1.00 (reference)
	Middle	55	126,043	43.6	0.70 (0.50, 0.98)	0.75 (0.53, 1.06)	0.75 (0.54, 1.06)
	High	35	127,700	27.4	0.56 (0.31, 0.68)	0.48 (0.32, 0.73)	0.49 (0.32, 0.74)
	*P for linear trend*				< 0.0001	< 0.0001	0.001
	Per 1-SD increase in cardiorespiratory fitness				0.45 (0.34, 0.59)	0.62 (0.48, 0.80)	0.62 (0.48, 0.80)
	Categories of grip strength						
	Low (reference)	81	136,899	59.2	1.00 (reference)	1.00 (reference)	1.00 (reference)
	Middle	54	124,095	43.5	0.75 (0.53, 1.06)	0.83 (0.58, 1.19)	0.83 (0.58, 1.18)
	High	42	118,689	35.4	0.61 (0.42, 0.89)	0.73 (0.49, 1.09)	0.71 (0.48, 1.06)
	*P for linear trend*				0.009	0.111	0.090
	Per 1-SD increase in grip strength				0.83 (0.71, 0.97)	0.90 (0.76, 1.06)	0.89 (0.76, 1.05)
Cancer mortality		503	379,682	132.5			
	Categories of cardiorespiratory fitness						
	Low (reference)	214	125,940	169.9	1.00 (reference)	1.00 (reference)	1.00 (reference)
	Middle	152	126,043	120.6	0.77 (0.62, 0.95)	0.76 (0.62, 0.94)	0.76 (0.61, 0.94)
	High	137	127,700	107.3	0.74 (0.60, 0.93)	0.72 (0.58, 0.90)	0.72 (0.58, 0.90)
	*P for linear trend*				0.006	0.003	0.003
	Per 1-SD increase in cardiorespiratory fitness				0.75 (0.64, 0.89)	0.80 (0.68, 0.94)	0.80 (0.68, 0.94)
	Categories of grip strength						
	Low (reference)	199	136,899	145.4	1.00 (reference)	1.00 (reference)	1.00 (reference)
	Middle	163	124,095	131.4	0.93 (0.75, 1.14)	0.94 (0.76, 1.16)	0.94 (0.76, 1.16)
	High	141	118,689	118.8	0.88 (0.71, 1.10)	0.91 (0.72, 1.14)	0.90 (0.72, 1.13)
	*P for linear trend*				0.259	0.393	0.352
	Per 1-SD increase in grip strength				1.00 (0.91, 1.10)	1.02 (0.92, 1.12)	1.01 (0.92, 1.12)

All models used age as the underlying time variable. Categories of aerobic fitness and grip strength were defined based on age and sex specific-categories of the baseline distribution. Aerobic fitness and grip strength were both normalized by fat-free mass

Model 1: No adjustment

Model 2: Adjusted for sex, waist circumference, ethnicity (White, mixed, Asian/Asian British, Black/Black British, other), smoking status (never, previous, current), employment (unemployed, employed), Townsend Deprivation Index, alcohol consumption (never, previous, currently < 3 times/week, currently ≥ 3 times/week), processed/red meat consumption (days/week), beta-blocker use, hypertension, and diabetes

Model 3: Model 2 plus grip strength in models where cardiorespiratory fitness was the exposure, or cardiorespiratory fitness in models where grip strength was the exposure

*CVD* cardiovascular disease, *SD* standard deviation

Compared with the lowest category of GS, the highest category had a significant HR of 0.80 (95% CI 0.67–0.96) while the HR for the middle category (HR 0.88; 95% CI 0.75–1.04) was not statistically significant after adjustment for confounders. Nonetheless, the linear trend across the three groups was significant (*p* for trend: 0.014). Additional adjustment for CRF made no meaningful differences to the associations (*p* for trend: 0.010). The HR of all-cause mortality for every 1-standard deviation increase in GS was 0.93 (95% CI 0.86–1.01), which was weaker than that for CRF.

Higher CRF was associated with lower hazards of CVD (*p* for trend: 0.001) and cancer (*p* for trend: 0.003) mortality, compared with the lowest category, after adjustment for confounders and GS. The highest CRF category had 51% (95% CI 26–68%) and 28% (95% CI 10–42%) lower hazards of CVD and cancer mortality, respectively, compared with the lowest category. CVD and cancer mortality rates were also lower in higher categories of GS, although the HRs were not statistically significant. Nonetheless, associations were stronger for CVD mortality compared to all-cause mortality. The HRs comparing CRF categories were larger than those for GS for all three mortality outcomes. Similar findings were identified in sensitivity analyses where CRF and GS were both normalized for body weight (S3 Table in Appendix), and deaths within the first 2-years of follow-up were excluded (S4 Table in Appendix).

**Fig. 2:**
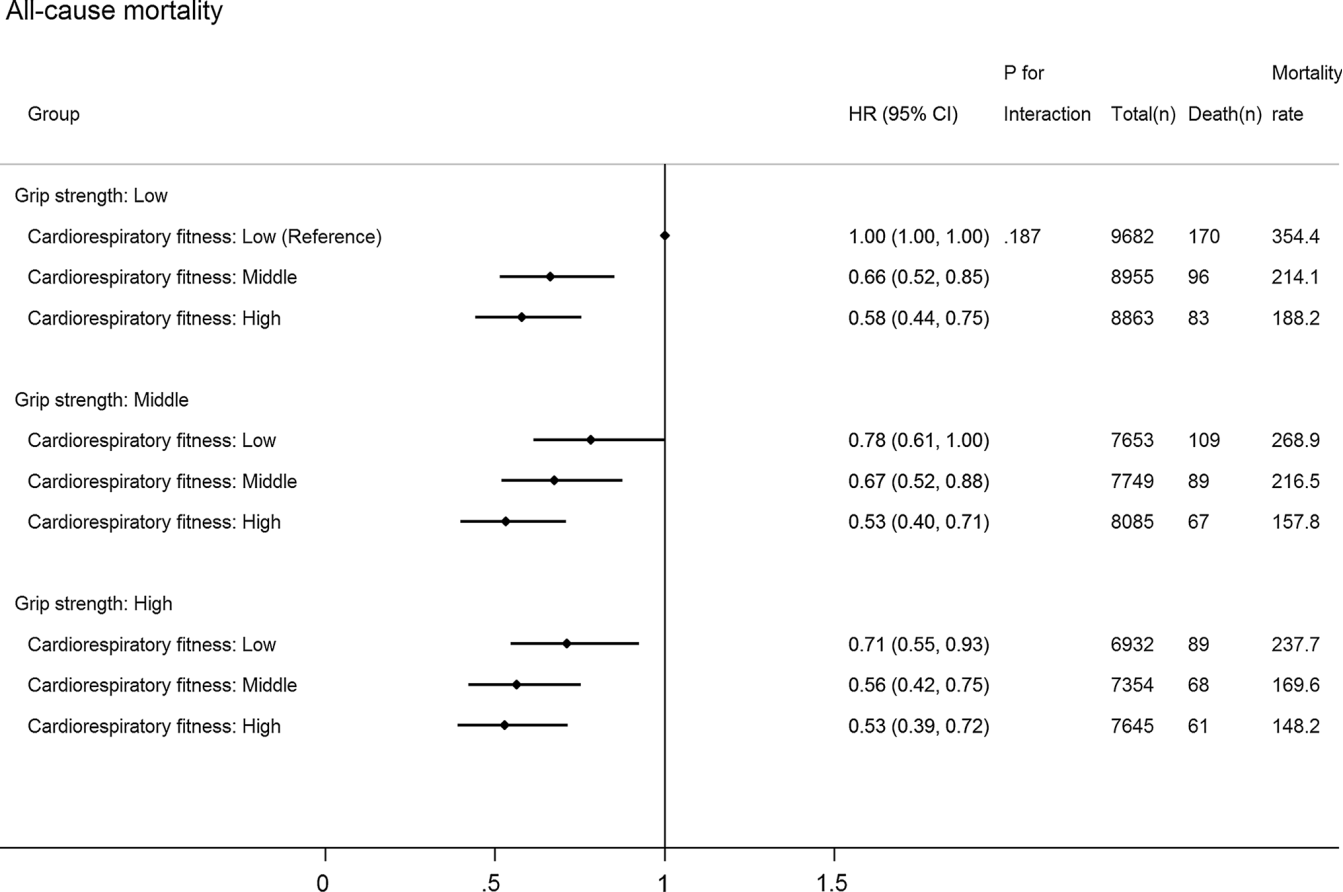
Joint associations of aerobic fitness and grip strength with all-cause mortality. The model was adjusted for sex, waist circumference, ethnicity (White, mixed, Asian/Asian British, Black/Black British, others), smoking status (never, previous, current), employment (unemployed, employed), Townsend Deprivation Index, alcohol consumption (never, previous, currently < 3 times/week, currently ≥ 3 times/week), processed/red meat consumption (days/week), beta-blocker use, hypertension, and diabetes. Age- and sex-specific categories of aerobic fitness and grip strength were used. Aerobic fitness and grip strength were both normalized by fat-free mass

**Fig. 3:**
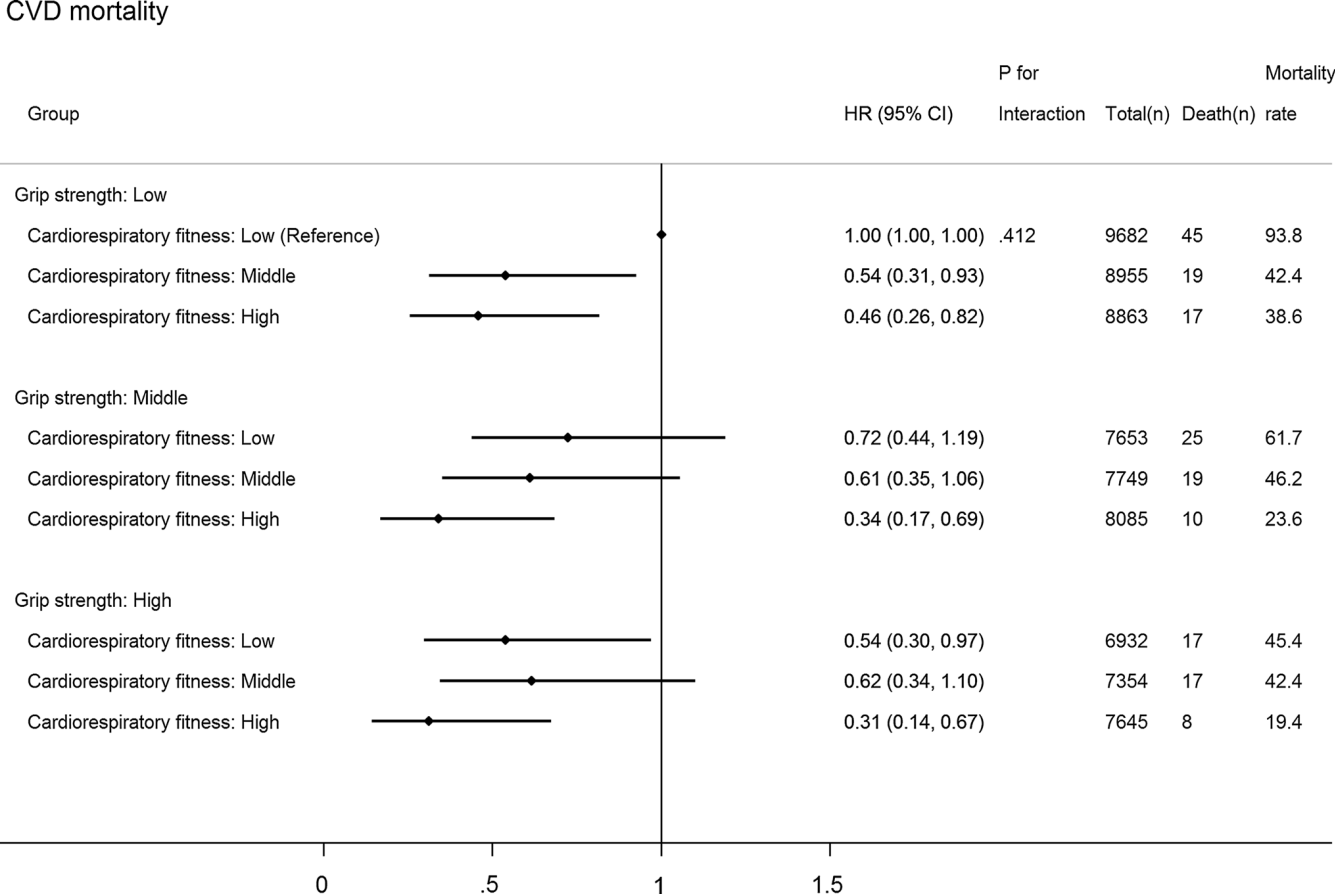
Joint associations of cardiorespiratory fitness and grip strength with cardiovascular disease (CVD) mortality. The model was adjusted for sex, waist circumference, ethnicity (White, mixed, Asian/Asian British, Black/Black British, others), smoking status (never, previous, current), employment (unemployed, employed), Townsend Deprivation Index, alcohol consumption (never, previous, currently < 3 times/week, currently ≥ 3 times/week), processed/red meat consumption (days/week), beta-blocker use, hypertension, and diabetes. Age- and sex-specific categories of cardiorespiratory fitness and grip strength were used. Cardiorespiratory fitness and grip strength were both normalized by fat-free mass

**Fig. 4:**
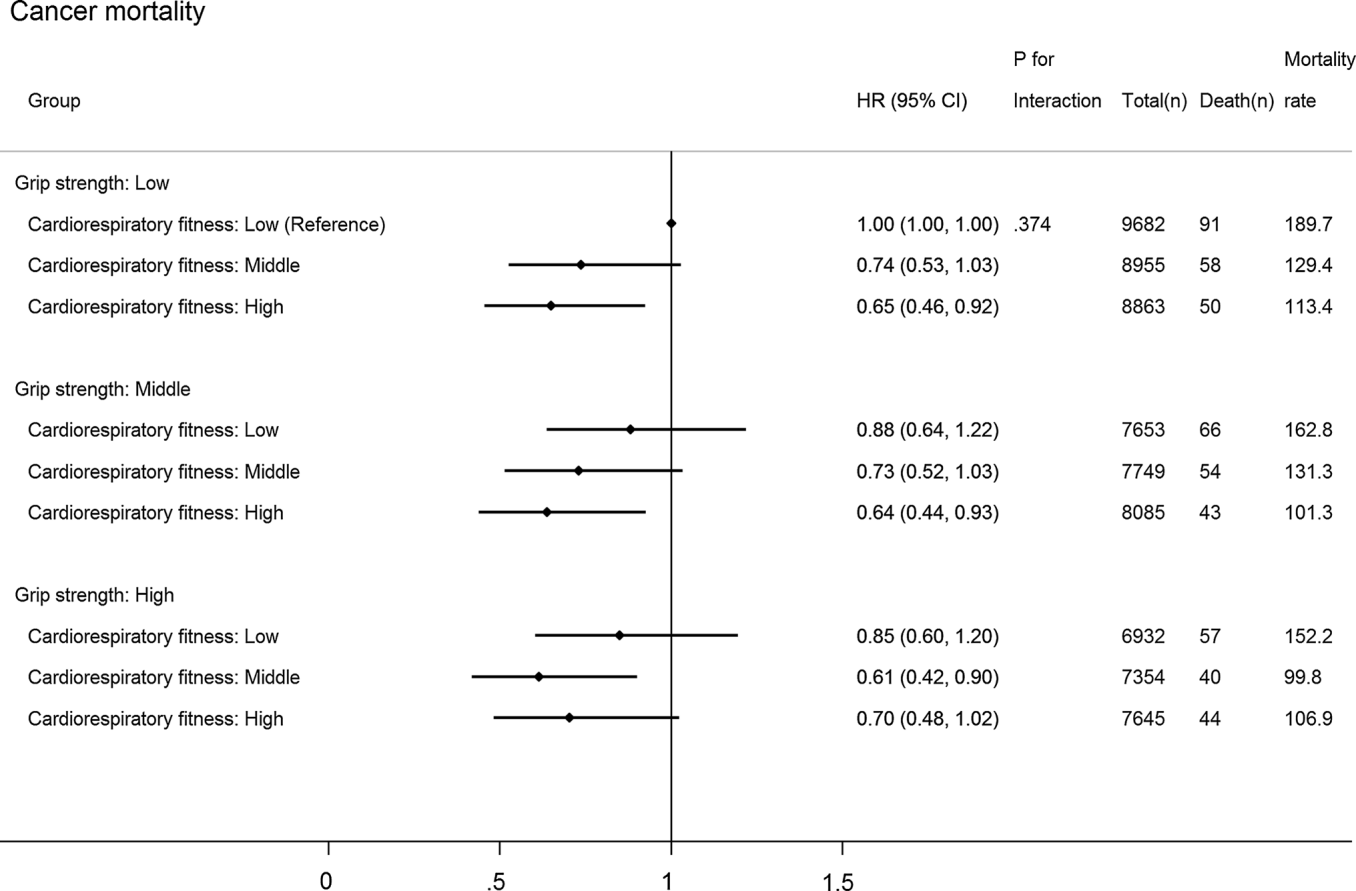
Joint associations of cardiorespiratory fitness and grip strength with cancer mortality. The model was adjusted for sex, waist circumference, ethnicity (White, mixed, Asian/Asian British, Black/Black British, others), smoking status (never, previous, current), employment (unemployed, employed), Townsend Deprivation Index, alcohol consumption (never, previous, currently < 3 times/week, currently ≥ 3 times/week), processed/red meat consumption (days/week), beta-blocker use, hypertension, and diabetes. Age- and sex-specific categories of cardiorespiratory fitness and grip strength were used. Cardiorespiratory fitness and grip strength were both normalized by fat-free mass

Figure 2 shows joint associations between CRF, GS and all-cause mortality (*p* for interaction: 0.187). Compared with individuals with the lowest CRF and GS, those with higher levels of both CRF and GS had lower hazards of all-cause mortality. The HR (compared with low GS and low CRF) in the highest CRF but lowest GS group (HR 0.58; 95% CI 0.44–0.75) was stronger than that in the lowest CRF but highest GS group (HR 0.71; 95% CI 0.55–0.93), although the 95% CIs around these two estimates overlapped. Compared with those with the lowest CRF and GS, individuals in the highest category of CRF and GS had a 47% (95% CI 28–61%) lower hazard of all-cause mortality and a 69% (95% CI 33–86%) lower hazard of CVD (Fig. 3; *p* for interaction: 0.412): no significant association for cancer mortality (HR 0.70; 95% CI 0.48–1.02) (Fig. 4; *p* for interaction: 0.374).

## Discussion

This study is the first investigation evaluating the relative risk of all-cause, CVD and cancer mortality for CRF, muscle strength and the combination of both in a general population of middle-aged and older men and women. Higher CRF was associated with lower risks of all-cause, CVD and cancer mortality, independent of GS and confounders. The highest GS was associated with lower risk of all-cause mortality compared with the lowest. Individuals with the highest level of CRF and GS had the lowest risks of all-cause and CVD mortality among all comparisons, compared with the lowest level of CRF and GS. The inverse associations were more consistent for higher CRF categories across GS levels than for higher GS categories across CRF levels.

Similar analyses have been reported in prior investigations but in highly selected populations [15, 16]. Using data from 1506 middle-aged hypertensive men, Artero et al. [15] found that compared with men in the lower 50% CRF group and the lowest muscle strength tertile, those in the upper 50% CRF group and the highest muscle strength tertile had a 51% lower risk of all-cause mortality. Similarly, a study of 1.5 million adolescent boys found that the lowest tertiles of CRF and muscle strength during adolescence were associated with more than twice the risk of all-cause (HR 2.01) and CVD (HR 2.63) mortality in later adulthood, compared with the highest tertiles [16]. Our study used data of men and women aged > 40 years with no critical medical conditions at baseline, and therefore provides new insights into the beneficial combined impacts of CRF and muscle strength on mortality risk in the general adult population.

The relatively stronger mortality associations with CRF than with GS suggest that lower CRF is a more important risk factor for mortality than lower muscle strength. A potential explanation for this difference is that the degree to which grip strength represents overall muscle strength may be lesser than the degree to which submaximal bike tests represent cardiorespiratory fitness. However, this finding is in line with prior research that found no significant [29] or inconsistent [30] associations of muscle strength (e.g., GS, sit-ups and push-ups [29]; bench press, leg press and sit-ups [30]) with mortality when CRF (e.g., predicted VO2max [29]; maximal-treadmill test [30]) was adjusted for in the analyses. Nonetheless, other studies using data of men concluded that muscle strength (e.g., bench press and leg press) was a strong predictor of mortality independent of CRF (e.g., maximal treadmill test) [31, 32]. In other studies, the combination of low CRF and muscle strength was associated with increased risks of developing stroke [33], type 2 diabetes [34], cardiovascular events and arrhythmia [35], all of which are strong mortality risk factors [36].

In addition to the evidence from observational studies, numerous intervention studies have demonstrated the synergistic effects of combining resistance training and aerobic exercise on eliciting favorable changes in intermediate health indicators. A recent 26-week randomized controlled trial of dieting obese older adults [9] found that individuals who received an intervention consisting of both aerobic and resistance exercise (3 days/week; 75–90 min each) plus a weight-management program showed relatively larger improvements in functional status and body composition in comparison with individuals who, in conjunction with a weight-management program, carried out either aerobic (3 days/week; 60 min each) or resistance exercise alone (3 days/week; 60 min each). Moreover, in a 9-month randomized-controlled trial of individuals with type 2 diabetes [10], hemoglobin A_1c_ levels significantly decreased in the intervention group who undertook a combined program of resistance training (2 days/week) and aerobic exercise (expending 10 kcal/kg/week) compared with the control group. Notably, this effect was not observed in the other intervention groups who received either resistance training (3 days/week) or aerobic exercise (expending 12 kcal/kg/week) [10]. Similarly, in an 8-month randomized controlled trial of 196 overweight adults aged 18–70 years [11], a combined protocol of resistance (3 days/week) and aerobic training (running 12 miles/week) resulted in significant improvements in insulin sensitivity, which was not achieved with either resistance or aerobic training alone. While clinical trials are needed to formally determine causality for the joint effects of CRF and muscle strength on mortality risk, these would be difficult to undertake in the general population; therefore, for the foreseeable future, public health action has to be informed by the combined evidence from exercise trials on intermediate risk factors and prospective observational epidemiological studies on clinical endpoints.

The current physical activity guidelines [17] recommend that adults do both moderate-to-vigorous intensity aerobic physical activity for 150 min/week and muscle-strengthening activities at least twice a week. Previous research found that meeting the guidelines for muscle-strengthening activities in addition to aerobic physical activity was associated with further reductions in the risks of comorbidity [37] and mortality [38]. Nonetheless, fewer than 30% of UK [39] and US [40] adults meet both the aerobic physical activity and muscle-strengthening guidelines. Furthermore, the prevalence of meeting these guidelines declines drastically with age [41]. Public health efforts should, therefore, be focused on encouraging adults of all ages to engage in both aerobic and resistance exercise to reduce mortality risk through increased CRF and muscle strength.

The following limitations should be considered when interpreting the findings. First, the findings of this study may not be generalizable to the whole UK population or adults in other countries as no sampling strategies were used in UK Biobank to select representative samples of adults. Another potential selection bias may exist with the sub-sample of individuals who performed bike tests. However, the UK Biobank employed less rigorous pre-test screening procedures compared with prior studies [15, 16], and those individuals who performed bike tests had virtually identical demographic and biological characteristics (e.g. sex ratio, GS, fat-free mass, resting pulse rate) to those who did not perform bike tests. In addition, there is risk of residual confounding due to the use of self-reported information (e.g., behaviors and comorbidities). Moreover, the findings may not be applicable to individuals with cancer, stroke or heart attack as these prevalent medical conditions were excluded from the present analyses. Furthermore, we may not have full follow-up information on individuals who migrated to other countries after participation in baseline assessment. Another limitation is the inability to draw firm conclusions about causal relationships of CRF and GS with mortality due to the observational nature of this study.

## Conclusions

Individuals with higher CRF showed lower risks of all-cause, CVD and cancer mortality; those with higher GS had lower all-cause mortality. All-cause and CVD mortality risk was lowest in adults with both higher CRF and higher. Improving both CRF and muscle strength, as opposed to either of the two alone, may be the most effective behavioral strategy to reduce all-cause and cardiovascular mortality risk.

## Electronic supplementary material

Below is the link to the electronic supplementary material.

Supplementary material 1 (DOCX 472 kb)
