# Supplementary material for: The combination of cardiorespiratory fitness and muscle strength, and mortality risk
Source: Eur J Epidemiol. 2018 Mar 28;33(10):953–64. doi: 10.1007/s10654-018-0384-x (PMC6153509; doi:10.1007/s10654-018-0384-x)
Supplement: Supplementary file 1 — Supplementary material 1 (DOCX 472 kb) [file 10654_2018_384_MOESM1_ESM.docx]

S1 Table. Age- and sex-specific cut-points used to create categories of cardiorespiratory fitness and grip strength

|  |  | Cardiorespiratory fitness (watts) per fat-free mass (kg) |  | Grip strength (kg) per fat-free mass (kg) |  |
| --- | --- | --- | --- | --- | --- |
| Age range | Category | Men | Women | Men | Women |
| <50yrs | Low | <3.75 | <3.25 | <0.59 | <0.51 |
|  | Middle | 3.75-4.55 | 3.25-4.11 | 0.59-0.70 | 0.51-0.62 |
|  | High | >4.55 | >4.11 | >0.70 | >0.62 |
|  |  |  |  |  |  |
| 50-59yrs | Low | <3.46 | <2.90 | <0.56 | <0.47 |
|  | Middle | 3.46-4.31 | 2.90-3.71 | 0.56-0.67 | 0.47-0.58 |
|  | High | >4.31 | >3.71 | >0.67 | >0.58 |
|  |  |  |  |  |  |
| ≥60yrs | Low | <2.99 | <2.37 | <0.54 | <0.44 |
|  | Middle | 2.99-3.90 | 2.37-3.12 | 0.54-0.64 | 0.44-0.54 |
|  | High | >3.90 | >3.12 | >0.64 | >0.54 |

S2 Table. Associations of cardiorespiratory fitness with mortality as estimated using a random-effects meta-analysis across different protocol assignments.

| Mortality type | Comparisons | Hazard ratios (95% confidence interval) |
| --- | --- | --- |
| **All-cause mortality** |  |  |
|  | **Categories of cardiorespiratory fitness** |  |
|  | Low (Reference) | 1.00 (Reference) |
|  | Middle | 0.68 (0.55, 0.81) |
|  | High | 0.70 (0.56, 0.83) |
|  |  |  |
| **CVD mortality** |  |  |
|  | **Categories of cardiorespiratory fitness** |  |
|  | Low (Reference) | 1.00 (Reference) |
|  | Middle | 0.66 (0.33, 0.98) |
|  | High | 0.49 (0.19, 0.78) |
|  |  |  |
| **Cancer mortality** |  |  |
|  | **Categories of cardiorespiratory fitness** |  |
|  | Low (Reference) | 1.00 (Reference) |
|  | Middle | 0.72 (0.54, 0.89) |
|  | High | 0.67 (0.50, 0.85) |

Note: Prior to performing the sub-maximal bike test in UK Biobank, each participant in the ‘minimal risk’ or ‘small risk’ category was allocated to one of 20 different ramp protocols (11 for men and 9 for women) based on their predicted maximal workload, which was a function of age, sex, weight, height and resting heart rate. In order to investigate the potential impacts of protocol allocation on associations between fitness and mortality, meta-analyses with random effects were performed that used combined categories of protocol assignments as follows in an attempt to preserve statistical power: a combined category of M40, M50, M60 and M70, a combined category of M80, M90 and M100, a combined category of M110, M120, M130 and M140, a combined category of F30, F40 and F50, a combined category of F60 and F70, and a combined category of F80, F90, F100 and F110. (M - males; F – females; numbers representing the peak power that was to be achieved by the end of the ramp test).

Models were adjusted for sex, waist circumference, ethnicity (White, mixed, Asian/Asian British, Black/Black British, other), smoking status (never, previous, current), employment (unemployed, employed), Townsend Deprivation Index, alcohol consumption (never, previous, currently <3times/week, currently ≥3times/week), processed/red meat consumption (days/week), beta-blocker use, hypertension, diabetes and grip strength.

S3 Table. Independent associations of weight-adjusted cardiorespiratory fitness (i.e. maximal workload divided by body weight) and weight-adjusted grip strength (i.e. grip strength divided by body weight) with all-cause, cardiovascular disease (CVD) and cancer mortality.

|  |  | Hazard Ratios (95% confidence interval) | | |
| --- | --- | --- | --- | --- |
| Mortality type | Comparisons | Model 1 | Model 2 | Model 3 |
| **All-cause**  **mortality** |  |  |  |  |
|  | **Categories of cardiorespiratory fitness** |  |  |  |
|  | Low (Reference) | 1.00 (Reference) | 1.00 (Reference) | 1.00 (Reference) |
|  | Middle | 0.78 (0.67, 0.92) | 0.80 (0.68, 0.94) | 0.80 (0.68, 0.94) |
|  | High | 0.70 (0.59, 0.83) | 0.72 (0.61, 0.87) | 0.73 (0.61, 0.87) |
|  | *P for linear trend* | <0.0001 | <0.0001 | <0.0001 |
|  | **Per 1-SD increase in cardiorespiratory fitness** | 0.69 (0.61, 0.79) | 0.79 (0.70, 0.90) | 0.80 (0.71, 0.90) |
|  |  |  |  |  |
|  | **Categories of grip strength** |  |  |  |
|  | Low (Reference) | 1.00 (Reference) | 1.00 (Reference) | 1.00 (Reference) |
|  | Middle | 0.82 (0.70, 0.97) | 0.87 (0.74, 1.03) | 0.87 (0.74, 1.03) |
|  | High | 0.81 (0.68, 0.96) | 0.89 (0.74, 1.08) | 0.89 (0.74, 1.07) |
|  | *P for linear trend* | 0.010 | 0.204 | 0.192 |
|  | **Per 1-SD increase in grip strength** | 1.40 (1.05, 1.87) | 0.80 (0.54, 1.19) | 0.80 (0.54, 1.18) |
|  |  |  |  |  |
| **CVD mortality** |  |  |  |  |
|  | **Categories of cardiorespiratory fitness** |  |  |  |
|  | Low (Reference) | 1.00 (Reference) | 1.00 (Reference) | 1.00 (Reference) |
|  | Middle | 0.54 (0.38, 0.77) | 0.60 (0.41, 0.86) | 0.60 (0.42, 0.87) |
|  | High | 0.49 (0.34, 0.72) | 0.57 (0.38, 0.85) | 0.58 (0.39, 0.86) |
|  | *P for linear trend* | <0.0001 | 0.003 | 0.003 |
|  | **Per 1-SD increase in cardiorespiratory fitness** | 0.43 (0.33, 0.57) | 0.63 (0.48, 0.81) | 0.63 (0.49 0.82) |
|  |  |  |  |  |
|  | **Categories of grip strength** |  |  |  |
|  | Low (Reference) | 1.00 (Reference) | 1.00 (Reference) | 1.00 (Reference) |
|  | Middle | 0.93 (0.66, 1.31) | 1.11 (0.78, 1.59) | 1.12 (0.78, 1.60) |
|  | High | 0.63 (0.42, 0.93) | 0.86 (0.56, 1.34) | 0.85 (0.55, 1.33) |
|  | *P for linear trend* | 0.024 | 0.596 | 0.569 |
|  | **Per 1-SD increase in grip strength** | 1.41 (0.75, 2.64) | 0.68 (0.29, 1.63) | 0.67 (0.28, 1.61) |
|  |  |  |  |  |
| **Cancer mortality** |  |  |  |  |
|  | **Categories of cardiorespiratory fitness** |  |  |  |
|  | Low (Reference) | 1.00 (Reference) | 1.00 (Reference) | 1.00 (Reference) |
|  | Middle | 0.85 (0.69, 1.05) | 0.84 (0.68, 1.04) | 0.84 (0.68, 1.04) |
|  | High | 0.79 (0.63, 0.98) | 0.78 (0.62, 0.98) | 0.78 (0.62, 0.98) |
|  | *P for linear trend* | 0.031 | 0.031 | 0.030 |
|  | **Per 1-SD increase in cardiorespiratory fitness** | 0.76 (0.64, 0.89) | 0.81 (0.69, 0.95) | 0.81 (0.69, 0.95) |
|  |  |  |  |  |
|  | **Categories of grip strength** |  |  |  |
|  | Low (Reference) | 1.00 (Reference) | 1.00 (Reference) | 1.00 (Reference) |
|  | Middle | 0.83 (0.67, 1.03) | 0.85 (0.68, 1.07) | 0.85 (0.68, 1.07) |
|  | High | 0.90 (0.72, 1.11) | 0.94 (0.74, 1.19) | 0.93 (0.73, 1.19) |
|  | *P for linear trend* | 0.274 | 0.551 | 0.538 |
|  | **Per 1-SD increase in grip strength** | 1.53 (1.05, 2.22) | 1.15 (0.69, 1.93) | 1.15 (0.69, 1.92) |

Note: All models used age as the underlying time variable. Categories of cardiorespiratory fitness and grip strength were defined based on age and sex specific-categories of the baseline distribution. Abbreviations: CVD – cardiovascular disease; SD – standard deviation.

Model 1: No adjustment.

Model 2: Adjusted for sex, waist circumference, ethnicity (White, mixed, Asian/Asian British, Black/Black British, other), smoking status (never, previous, current), employment (unemployed, employed), Townsend Deprivation Index, alcohol consumption (never, previous, currently <3times/week, currently ≥3times/week), processed/red meat consumption (days/week), beta-blocker use, hypertension, and diabetes.

Model 3: Model 2 plus cardiorespiratory fitness in models where grip strength was the exposure, or grip strength in models where cardiorespiratory fitness was the exposure.

S4 Table. Independent associations of cardiorespiratory fitness and grip strength with all-cause, cardiovascular disease (CVD) and cancer mortality after excluding 195 mortality cases accrued during the first 2-year follow-up period.

|  |  | Hazard Ratios (95% confidence interval) | | |
| --- | --- | --- | --- | --- |
| Mortality type | Comparisons | Model 1 | Model 2 | Model 3 |
| **All-cause**  **mortality** |  |  |  |  |
|  | **Categories of cardiorespiratory fitness** |  |  |  |
|  | Low (Reference) | 1.00 (Reference) | 1.00 (Reference) | 1.00 (Reference) |
|  | Middle | 0.79 (0.65, 0.95) | 0.79 (0.65, 0.95) | 0.79 (0.66, 0.95) |
|  | High | 0.76 (0.63, 0.93) | 0.75 (0.62, 0.91) | 0.75 (0.62, 0.92) |
|  | *P for linear trend* | 0.004 | 0.003 | 0.003 |
|  | **Per 1-SD increase in cardiorespiratory fitness** | 0.74 (0.64, 0.85) | 0.81 (0.71, 0.93) | 0.81 (0.71, 0.93) |
|  |  |  |  |  |
|  | **Categories of grip strength** |  |  |  |
|  | Low (Reference) | 1.00 (Reference) | 1.00 (Reference) | 1.00 (Reference) |
|  | Middle | 0.90 (0.75, 1.07) | 0.93 (0.78, 1.12) | 0.93 (0.77, 1.12) |
|  | High | 0.74 (0.61, 0.90) | 0.80 (0.65, 0.98) | 0.80 (0.65, 0.98) |
|  | *P for linear trend* | 0.003 | 0.037 | 0.032 |
|  | **Per 1-SD increase in grip strength** | 0.92 (0.85, 0.99) | 0.95 (0.87, 1.04) | 0.95 (0.87, 1.04) |
|  |  |  |  |  |
| **CVD mortality** |  |  |  |  |
|  | **Categories of cardiorespiratory fitness** |  |  |  |
|  | Low (Reference) | 1.00 (Reference) | 1.00 (Reference) | 1.00 (Reference) |
|  | Middle | 0.88 (0.61, 1.29) | 0.94 (0.64, 1.37) | 0.94 (0.64, 1.38) |
|  | High | 0.57 (0.36, 0.88) | 0.58 (0.37, 0.92) | 0.58 (0.37, 0.92) |
|  | *P for linear trend* | 0.014 | 0.026 | 0.027 |
|  | **Per 1-SD increase in cardiorespiratory fitness** | 0.51 (0.37, 0.69) | 0.68 (0.52, 0.89) | 0.68 (0.52, 0.90) |
|  |  |  |  |  |
|  | **Categories of grip strength** |  |  |  |
|  | Low (Reference) | 1.00 (Reference) | 1.00 (Reference) | 1.00 (Reference) |
|  | Middle | 0.80 (0.54, 1.17) | 0.90 (0.60, 1.33) | 0.89 (0.60, 1.32) |
|  | High | 0.63 (0.41, 0.96) | 0.77 (0.49, 1.20) | 0.76 (0.48, 1.18) |
|  | *P for linear trend* | 0.029 | 0.252 | 0.221 |
|  | **Per 1-SD increase in grip strength** | 0.85 (0.71, 1.01) | 0.93 (0.77, 1.12) | 0.92 (0.77, 1.11) |
|  |  |  |  |  |
| **Cancer mortality** |  |  |  |  |
|  | **Categories of cardiorespiratory fitness** |  |  |  |
|  | Low (Reference) | 1.00 (Reference) | 1.00 (Reference) | 1.00 (Reference) |
|  | Middle | 0.75 (0.59, 0.96) | 0.74 (0.58, 0.94) | 0.73 (0.57, 0.94) |
|  | High | 0.83 (0.65, 1.06) | 0.80 (0.62, 1.02) | 0.79 (0.62, 1.02) |
|  | *P for linear trend* | 0.100 | 0.057 | 0.054 |
|  | **Per 1-SD increase in cardiorespiratory fitness** | 0.79 (0.66, 0.96) | 0.83 (0.69, 0.99) | 0.82 (0.69, 0.99) |
|  |  |  |  |  |
|  | **Categories of grip strength** |  |  |  |
|  | Low (Reference) | 1.00 (Reference) | 1.00 (Reference) | 1.00 (Reference) |
|  | Middle | 0.96 (0.76, 1.22) | 0.99 (0.77, 1.26) | 0.99 (0.77, 1.25) |
|  | High | 0.86 (0.67, 1.11) | 0.91 (0.70, 1.18) | 0.90 (0.69, 1.17) |
|  | *P for linear trend* | 0.250 | 0.477 | 0.448 |
|  | **Per 1-SD increase in grip strength** | 1.01 (0.90, 1.12) | 1.04 (0.92, 1.16) | 1.03 (0.92, 1.16) |

Note: All models used age as the underlying time variable. Categories of cardiorespiratory fitness and grip strength were defined based on age and sex specific-categories of the baseline distribution. Cardiorespiratory fitness and grip strength were both normalized by fat-free mass. Abbreviations: CVD – cardiovascular disease; SD – standard deviation.

Model 1: No adjustment.

Model 2: Adjusted for sex, waist circumference, ethnicity (White, mixed, Asian/Asian British, Black/Black British, other), smoking status (never, previous, current), employment (unemployed, employed), Townsend Deprivation Index, alcohol consumption (never, previous, currently <3times/week, currently ≥3times/week), processed/red meat consumption (days/week), beta-blocker use, hypertension, and diabetes.

Model 3: Model 2 plus cardiorespiratory fitness in models where grip strength was the exposure, or grip strength in models where cardiorespiratory fitness was the exposure.

S1 Material. Allocation of risk category.

| Risk Factor | Definition | Risk Category |
| --- | --- | --- |
| Heart condition | answer to “Has a doctor ever said that you have a heart condition and that you should only do physical activity recommended by a doctor” is ‘yes’ or ‘unsure’ | Small risk |
| Chest pain during physical activity | answer to “Do you feel pain in your chest when you do physical activity” is ‘yes’ or ‘unsure’ | Medium risk |
| Chest pain at rest | answer to “In the past month, have you had chest pain when you were NOT doing physical activity” is ‘yes’ or ‘unsure’ | High risk |
| Unable to walk/cycle | answer to “Are you able to walk or cycle unaided for 10 minutes” is ‘no’ or ‘unsure’ | High risk |
| Pregnant | participant is female and has not been declared as definitely not pregnant | High risk |
| Height unknown | participant's height is unknown | Medium risk |
| Weight unknown | participant's weight is unknown | High risk |
| Heart rate unknown | participant's resting heart rate is unknown | Medium risk |
| Blood pressure unknown | systolic or diastolic bp is unknown | High risk |
| Blood pressure very high | lowest systolic bp >= 180 or lowest diastolic bp >= 110 | High risk |
| Blood pressure high | lowest systolic bp >= 160 or lowest diastolic bp >= 95 | Small risk |
| Weight high | Weight >=150 kg | High risk |
| Pacemaker unknown | participant has not declared as not having pacemaker | High risk |
| Pacemaker | participant has pacemaker | Electrocardiogram to be avoided (either unsafe or pointless) |

Note: Participants without any of the risk factors were considered to have ‘minimal risk’. Participant with one or more risk factors are in the highest risk category corresponding to those risk factors. Information in the table was modified and/or copied from Appendix 1 of the UK Biobank Cardio Assessment protocol document [^1^](#_ENREF_1).

S2 Material. Prediction of absolute maximum workload.

The following equations were used to predict maximum workload before the bike test; this was then used to allocate each participant to an individualized bike protocol[^1^](#_ENREF_1):

For men,

Absolute Maximum Workload (watts) = 108.0844 + (-0.47184 × Age) + (-0.0280973 × Age × Age) + (179.7342 × Height) + (0.1593 × Weight) + (-3.07113 × Resting heart rate) + (0.0090985 × Resting heart rate × Resting heart rate) + (0.0180811 × Resting heart rate × Age)

For women,

Absolute Maximum Workload (watts) = 105.2749 + (-0.0935 × Age) + (-0.0280973 × Age × Age) + (119.0087 × Height) + (0.309456 × Weight) + (-2.698067 × Resting heart rate) + (0.0090985 × Resting heart rate × Resting heart rate) + (0.0180811 × Resting heart rate × Age)

Note: Age is in years, height is in meters, weight is in kilograms and resting heart rate is in beats per minute.

Data materials for deriving these prediction equations are based on 10,973 maximal bike tests from the Danish National Health Examination Survey (“DANHES”) 2007-2008.[^2^](#_ENREF_2)

S3 Material. Data processing procedures for cadence data from cardiorespiratory fitness assessments

- Participants were asked to cycle at 60 revolutions-per-minute (RPM) during all cycling phases. The workload (adaptive braking) of the bike was only guaranteed to be accurate by the manufacturer when cycling between 30 and 130 RPM; observations were excluded if cadence was outside the range of 35-125 RPM, as were observations in the 30 seconds subsequent to any such event. This procedure also limits variation in the internal power component. Premature cessation of cycling prior to the programmed onset of the recovery phase was detected as a sudden drop in cadence, resulting in re-annotation of test/recovery data.
- To correctly annotate the test phases (Test versus Recovery), the following algorithm was used:
  - - - 1. Examine if the last cadence value is less than the second-to-last cadence value (i.e. negative difference) within the Test phase
        2. Iterate this procedure over all cadence values of the Test phase (starting from the last cadence value of the Test phase)
        3. Stop this iteration procedure when the current cadence value is equal to or greater than the preceding cadence value (i.e. 0 or positive difference) within the Test phase.
        4. Define a time window of potential re-annotation as the time point corresponding to the cadence value equal to or greater than the preceding cadence in the iteration procedure (Steps (1)-(3)) through the time point corresponding to the very last cadence of the Test phase.

Within the identified time window of potential re-annotation, calculate the difference between the maximum and minimum cadence, and calculate the number of data entries captured.

If, within the time window of potential re-annotation, the difference in cadence is ≥ 5 RPM or the number of data entries ≥ 2, then re-annotate the end of the Test phase to the time corresponding to the cadence value equal to or greater than the preceding cadence in the iteration procedure (Steps (1)-(3)) (All time periods after this point in time were reclassified as a recovery phase)

If, within the time window of potential re-annotation, the difference in cadence is < 5 RPM or the number of data entries < 2, then retain the original phase annotation.

S4 Material. Procedures for deriving cardiorespiratory fitness using ECG data collected from sub-maximal bike tests


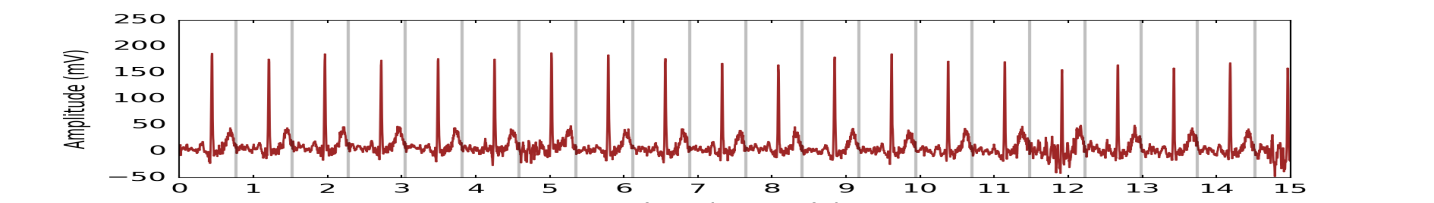


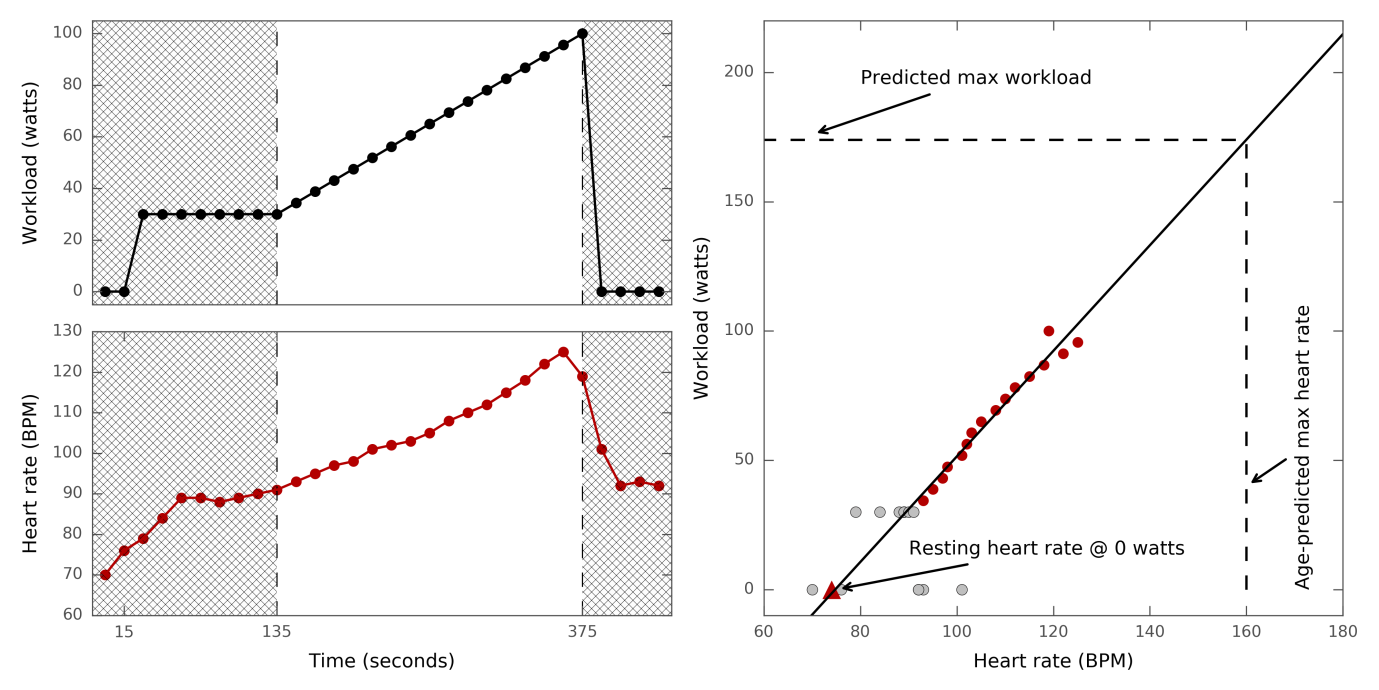


- The top panel in the above figure shows the beat detection algorithm on raw ECG for a 20-sec sample.
- The middle left panel shows instantaneous heart rate (beats-per-minute) as derived by peak detection algorithm and beat outlier filtering against time; the bottom left panel show and workload (bottom, watts) against time (15-sec pretest, 2-min constant load phase, 4-min ramp load protocol, and 1-min recovery phase).
- The right panel illustrates the regression-and-extrapolation procedure used to estimate cardiorespiratory fitness using paired data of heart rate and workload from the 4-min ramp load protocol (highlighted in red). Note: this is an artificial dataset created to illustrate the principle of inference.
  - - 1. A linear regression model was fit to these data plus a pair of resting pulse rate (measured during seated blood pressure measurements[^3^](#_ENREF_3)) and 0 watt in order to establish equations to predict workload from heart rate.
      2. The established linear relationship (i.e. beta coefficient and intercept) was then used to predict maximal workload (watts) from the participant’s age-predicted maximum heart rate, calculated using the equation of (208 – 0.7×Age).[^4^](#_ENREF_4)
- Linear regression models were also fit to data from the constant load phase: both the first 2-minute constant phase of the full 6-minute bike test in individuals allocated to a ramp load protocol and the 6-minute constant phase in individuals with no ramp allocation. In both cases, there was no ramp increase in workload during the test.
  1. Therefore, linear regression analyses were performed using two data points: resting pulse rate (measured during blood pressure measurements[^3^](#_ENREF_3)) and the average heart rate of the last 15 seconds of the minute constant phase, corresponding to 0 watt and constant power (30 watts for women; 40 watts for men), respectively.
  2. The established linear relationship (i.e. beta coefficients and intercepts) was then used to predict maximal workload (watts) based on age-predicted maximum heart rate, calculated using the equation of 208 – 0.7×Age.[^4^](#_ENREF_4)

S5 Material. Procedures for consolidating aerobic fitness estimates derived using different types of data sources from different types of bike test phases.


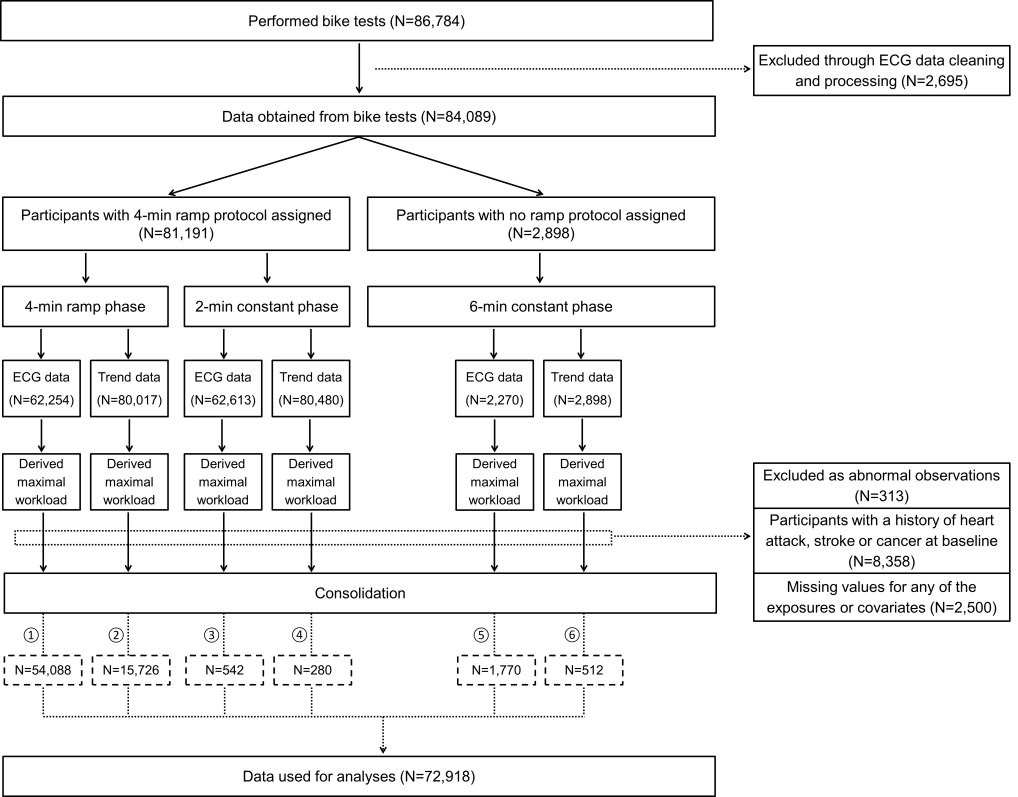


- The above flow diagram illustrates the pipelines through which different data sources from different bike test phases were processed in order to consolidate estimates of derived maximal workload.
- The bike protocol for individuals who were allocated to a ramp load protocol consisted of a 4-min ramp phase as well as a 2-min constant phase (30watts for women; 40watts for men). In contrast, individuals who were not allocated to a ramp protocol but a constant load protocol cycled at a constant workload level (30watts for women; 40watts for men) for full 6 minutes.
- Both the ramp and constant phase (either 2-min or 6-min) provided electrocardiograph (ECG) and “Trend” data, both of which were processed using the derivation procedures (Material 3 in Appendix) in order to derive a maximal workload value for each data source for each test phase. Note: Heart rate estimates from the lower resolution (approximately every 10-sec) “Trend” data were provided by the device’s on-board peak detection and filtering algorithm; our internal validation work found these heart rate estimates to be comparable to heart rate values from the ECG data.
- In terms of consolidating estimates of maximal workload, the order of preferred data sources is as follows:
  1. ECG data from the 4-min ramp phase,
  2. Trend data from the 4-min ramp phase,
  3. ECG data from the 2-min constant phase in participants with an allocated ramp protocol,
  4. Trend data from the 2-min constant phase in participants with an allocated ramp protocol,
  5. ECG data from the 6-min constant phase in participants with no ramp protocol, and
  6. Trend data from the 6-min constant phase in participants with no ramp protocol.
- For example, if the maximal workload value derived using ECG data from the 4-min ramp phase was unavailable or the corresponding beta coefficients for the relationship between workload and heart rate was negative (i.e. biologically implausible), then the maximal workload value derived using Trend data from the 4-min ramp phase was used instead. Similarly, if the maximal workload value derived using Trend data from the 4-min ramp phase was unavailable or the corresponding beta coefficients for the relationship between workload and heart rate was negative, then the maximal workload value derived using ECG data from the 2-min constant phase of the 6-min bike test was used instead.

**References**

**1.** UK Biobank: Cardio Assessment Version 1.0. 2011.

**2.** Eriksen L, Gronbaek M, Helge JW, Tolstrup JS. Cardiorespiratory fitness in 16 025 adults aged 18-91 years and associations with physical activity and sitting time. *Scand J Med Sci Sports.* 2016;26(12):1435-1443.

**3.** UK Biobank Coordinating Centre. UK Biobank: Protocol for a large-scale prospective epidemiological resource. *Design.* 2007:06: 01-112.

**4.** Tanaka H, Monahan KD, Seals DR. Age-predicted maximal heart rate revisited. *J Am Coll Cardiol.* 2001;37(1):153-156.
